# A Genetic Screen To Assess Dopamine Receptor (DopR1) Dependent Sleep Regulation in *Drosophila*

**DOI:** 10.1534/g3.116.032136

**Published:** 2016-10-18

**Authors:** Yiqin Jiang, Elise Pitmon, Jack Berry, Fred W. Wolf, Zach McKenzie, Tim J. Lebestky

**Affiliations:** * Department of Biology, Williams College, Williamstown, Massachusetts 01267; † University of California, School of Natural Sciences Merced, California 95343

**Keywords:** arousal, dopamine, *Drosophila*, mushroom body, sleep

## Abstract

Sleep is an essential behavioral state of rest that is regulated by homeostatic drives to ensure a balance of sleep and activity, as well as independent arousal mechanisms in the central brain. Dopamine has been identified as a critical regulator of both sleep behavior and arousal. Here, we present results of a genetic screen that selectively restored the Dopamine Receptor (*DopR/DopR1/dumb*) to specific neuroanatomical regions of the adult *Drosophila* brain to assess requirements for DopR in sleep behavior. We have identified subsets of the mushroom body that utilizes DopR in daytime sleep regulation. These data are supported by multiple examples of spatially restricted genetic rescue data in discrete circuits of the mushroom body, as well as immunohistochemistry that corroborates the localization of DopR protein within mushroom body circuits. Independent loss of function data using an inducible RNAi construct in the same specific circuits also supports a requirement for DopR in daytime sleep. Additional circuit activation of discrete DopR^+^ mushroom body neurons also suggests roles for these subpopulations in sleep behavior. These conclusions support a new separable function for DopR in daytime sleep regulation within the mushroom body. This daytime regulation is independent of the known role of DopR in nighttime sleep, which is regulated within the Fan-Shaped Body (FSB). This study provides new neuroanatomical loci for exploration of dopaminergic sleep functions in *Drosophila*, and expands our understanding of sleep regulation during the day *vs.* night.

Identifying the cellular and molecular mechanisms that control arousal and sleep is an important pursuit for understanding rest homeostasis (Shaw *et al.* 2000), as well as facilitating a deeper understanding of sleep disorders in humans (Donelson and Sanyal 2015). In *Drosophila melanogaster*, sleep has been characterized as consolidated periods of rest marked by a decreased responsivity to arousing stimuli and represented as a homeostatic drive that requires fulfillment of rest for optimal performance in cognitive and innate tasks (Dissel *et al.* 2015). Sleep in *Drosophila* can be divided into multiple behavioral dimensions for investigation of potentially separable aspects of sleep behavior, such as onset of sleep, duration, number of sleep bouts, and average duration of individual sleep bouts. Furthermore, both genetic and environmental factors, such as temperature and light, can differentially affect parameters of *Drosophila* sleep in the day *vs.* night period (Ishimoto *et al.* 2012; Parisky *et al.* 2016).

Dopamine has been identified as a key regulator of sleep in *Drosophila* (Andretic and Hirsh 2000; Kume *et al.* 2005; Lebestky *et al.* 2009; Sitaraman *et al.* 2015b); however, the cellular loci of presynaptic and postsynaptic control are complex. Previously, a requirement for the Type I Dopamine Receptor, *DopR/DopR1/dumb*, was localized to the dorsal FSB, as well as an absence of DopR function in the mushroom body with regards to observed sleep behavior (Ueno *et al.* 2012). This contrasts with a previously known requirement for neural activity in the mushroom body in sleep behavior (Joiner *et al.* 2006; Pitman *et al.* 2006); however, it could be consistent with independent control of sleep behavior in the mushroom body that is not subject to direct dopaminergic regulation. Alternatively, multiple groups have recently ascribed microcircuitry, or discrete, functional subsets of neurons within the brain, as having opposing roles or separable functions within a larger structure, and this may also explain differences in broad *vs.* specific manipulations within a given neuroanatomical structure or class of neurons (Seidner *et al.* 2015; Sitaraman *et al.* 2015a).

Given the known regions of high DopR expression in discrete brain structures such as the central complex and mushroom body (Kim *et al.* 2003; Kong *et al.* 2010; Lebestky *et al.* 2009), and the potential for less conspicuous but functionally relevant DopR in other brain regions, we sought to utilize the dominant, haploinsufficient sleep phenotype of the UAS piggyBac insertional mutation, *DopR^f02676^*/+, heterozygous animals as a sensitized screening background for identifying new neural circuits that use DopR in regulating sleep behavior (Figure 1). This genotype has proven to be a useful tool in characterizing dopamine signaling and the circuit-based requirements for DopR in multiple *Drosophila* behaviors (Kim *et al.* 2007; Kong *et al.* 2010; Lebestky *et al.* 2009). Previous data suggests a clear excess of sleep for both *DopR^f02676^/+* and *DopR^f02676^/DopR^f02676^* mutant animals (Lebstky *et al.* 2009). In the following study, we utilized many neuronal GAL4 lines, as well as lines from the FlyLight collection of Gal4 lines (Jenett *et al.* 2012), to restore DopR in discrete circuits of the brain and CNS and assess changes to sleep behaviors. The highly anatomically characterized FlyLight Collection allows for improved specificity for individual Gal4 lines to allow for deeper investigation of DopR requirements in sleep behavior. Here, we report the screening procedures and results for identifying new neural circuits that require *DopR* for normal daytime sleep behavior. Furthermore, this day regulation is shown to be separable from the existing known requirement for *DopR* in nighttime sleep behavior.

**Figure 1:**
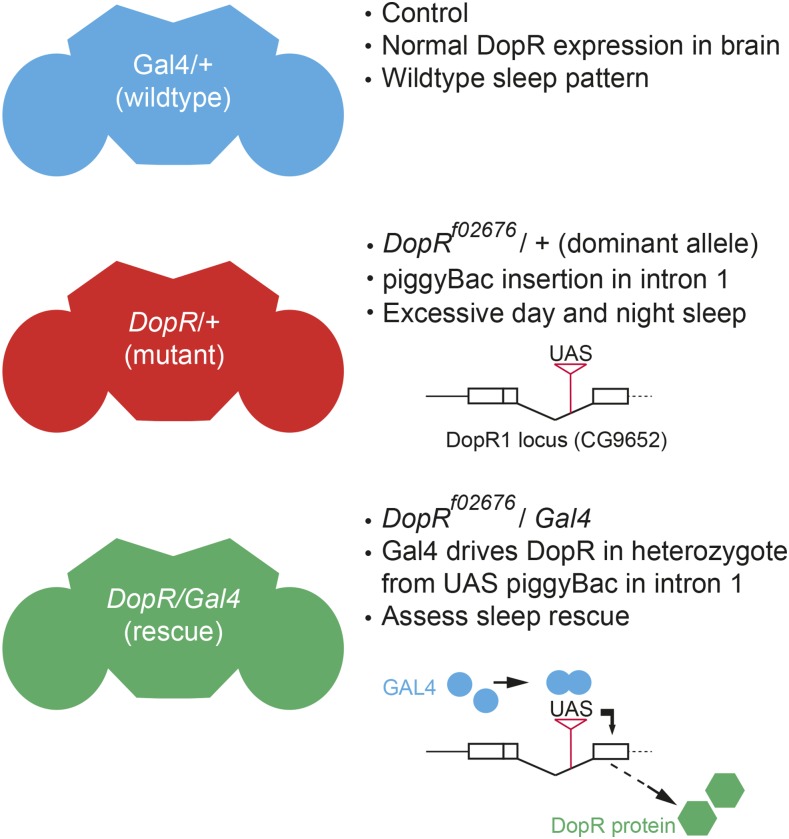
Schematic representation of screen conditions. Restoration of normal wild-type sleep and activity patterns is assessed relative to selective restoration of DopR function in subsets of neurons in the *Drosophila* adult brain. If sleep levels in the rescue genotype (*Gal4/DopR^f02676^*) are equivalent to wild-type behavior (*Gal4/+*) and significantly different from *DopR^f02676^*/+, as measured by one-way ANOVA comparison, the neuronal Gal4 line is regarded as a successful rescue. DopR, dopamine receptor; UAS, upstream activating sequence.

## Materials and Methods

### Genetic screen: F1 crosses for behavioral analysis

The dominant, hypomorphic mutant allele *DopR^f02676^* contains a *piggy-Bac* element insertion with a UAS sequence in the first intron (Exelixis Collection at Harvard Medical School). This allele displays haplo-insufficiency for certain behaviors, as we and others observe a loss of mRNA (50–60%) and reductions in protein expression (observed via immunohistochemistry) in the adult brain (Lebestky *et al.* 2009; Kong *et al.* 2010). When crossed to Gal4 stocks, the resultant genotype (*Gal4/+*;*DopR^f02676^/+*) will produce a truncated, but functional version of the protein only where the Gal4 is expressed (Lebestky *et al.* 2009; Kong *et al.* 2010; Kim *et al.* 2007). It is currently unknown whether the *DopR^f02676^* allele, alone in the absence of Gal4, also produces alternatively spliced or dominant negative forms of the protein. The *DopR^f02676^* allele was backcrossed into the Canton-S (*CS*) wild-type background for six generations (as described in Lebestky *et al.* 2009). Gal4 lines were acquired from the Kaiser collection (Armstrong *et al.* 1998) and the Janelia Farm FlyLight collection at Bloomington *Drosophila* Stock Center (BDSC; Bloomington, IN). Expression data including high resolution z-stack projections and movies for all FlyLight collection Gal4 lines are made available to the entire *Drosophila* community by HHMI Janelia Farm for review at the following URL: http://flweb.janelia.org/cgi-bin/flew.cgi. All lines used in our study can be observed by selecting “Line or associated gene (adult)” in the “Search Lines” function and inserting the genotype into the search window (R59H05 for example). Fly stocks were maintained at 18°, and crosses were grown at 25°. All flies were kept in 12:12 light:dark (LD) cycle conditions. Flies were reared on Bloomington recipe fly food. Fly strains generated in our lab are available for distribution and data sets for sleep parameters are available upon request.

For F1 screening, *DopR^f02676^* homozygous virgins or CS virgin females were crossed with homozygous *Gal4* males, and F1 males were selected. Genetic controls for *Gal4* flies acquired from the Janelia Farm collection consist of a recombination of control fly line that is “empty” at the recombination attp2 site at 68A4 on 3L (Jenett *et al.* 2012; Pfeiffer *et al.* 2008). Controls for Gal4 lines of the Kaiser collection (Armstrong *et al.* 1998) were performed by crossing CS virgins flies to males with the Gal4 enhancer trap c561, which has a *P*-element insert on the X chromosome and is therefore not transmitted to F1 hybrid males within the given crossing scheme, but autosomes represented in the Kaiser collection are present in the F1 hybrid.

Determination of whether a given Gal4-rescue genotype is regarded as a “fail” *vs.* “rescue” in Table 1 and Table 2 is based on the statistical comparison for total sleep and sleep duration between the Gal4/+ and DopR/Gal4 genotypes (See *Statistics* below). If these are not significantly different from each other and they are significantly different from DopR/+, the potential rescue condition is validated. If the rescue condition is not significantly different from the DopR/+ and is significantly different from the Gal4/+ genotype, the rescue genotype is regarded as a “fail.” For rare cases where Gal4/+ is not significantly different from DopR/+ for sleep parameters, indicating confounds due to insertional variation or genetic background, the line is also regarded as a “fail.”

**Table 1:** Neural circuits screened and expression patterns in adult *Drosophila* brain

Janelia FlyLight Gal4	Genomic Origin	Site(s) of Primary Expression	Sleep Rescue
R14HO4	CG9907	AL	Fail
R70E03	CG30361	AL	Fail
R73D06	CG9097	NOD	Fail
R83H12	CG1849	NOD	Fail
R53B06	CG16766	LH	Fail
R10B01	CG7664	OL	Fail
R17F12	CG3454	OL	Fail
R33H10	CG7524	OL	Fail
R20D05	CG9554	CAN	Fail
R21H12	CG5610	CAN	Fail
R27E02	CG32171	AMMC	Fail
R64C04	CG7395	AMMC	Fail
R83C03	CG1849	AMMC	Fail
R23C12	CG14307	PL PROTOCER	Fail
R22H09	CG2872	SOG	Fail
R26G08	CG10772	SOG	Fail
R27G01	CG32171	SOG	Fail
R32B04	CG10388	SOG, OL	Fail
R45H03	CG1429	AMMC, AL, MB	Fail
R59C12	CG31665	OL, AMMC, MB, EB	Fail
R59G03	CG7958	MB, FSB, OL	Fail
R75F05	CG4807	MB, PCB, LH	Fail
R37G12	CG1004	FSB, LH, PCB	Fail
R82G02	CG1133	MB, BULB, PCB	Fail
R21D08	CG6383	MB subset, LH, PROW	Fail
R59H07	CG32296	MB subset	Fail
R23D03	CG14307	MB, PCB, LH, SOG	Rescue
R87E08	CG3340	MB Subset	Rescue
R87B01	CG18389	MB subset	Rescue
R59H05	CG7467	MB subset	Rescue
Kaiser Collection			
c119	Unknown	EB	Fail
36y	Unknown	EB	Fail
95y	Unknown	EB	Fail
c232	Unknown	EB	Fail
201y	Unknown	MB	Fail
117y	Unknown	γ MB	Fail
43y	Unknown	MB	Fail
30y	Unknown	MB, FSB, EB, OL, PI, LH	Fail
c305a	Unknown	MB	Rescue
c5	Unknown	Dorsal FSB, MB	Rescue

“Sites of Primary Expression” is operationally defined as neuroanatomical regions that display highest expression by review of confocal stacks from Fly-Light expression studies. Abbreviations for brain regions expressing Gal4: AL, antennal lobe; NOD, noduli; LH, lateral horn; OL, optic lobe; CAN, cantle; AMMC, antennal mechanosensory and motor center; PL PROTOCER, posterior lateral protocerebrum; SOG, subesophageal ganglion; MB, mushroom body; FSB, fan-shaped body; EB, ellipsoid body; PCB, protocerebral bridge; PI, pars intercerebralis/median bundle.

**Table 2:** Screened Gal4 lines derived from DopR genomic enhancer elements (CG9652)

Janelia FlyLight Gal4	Genomic Origin	Site(s) of Primary Expression	Sleep Rescue
R72B09	CG9652	EB, LH, SOG, MB	Fail
R72B12	CG9652	MB, AL, FSB, NOD, BROAD LOW	Fail
R72C02	CG9652	MB, OL, AL, LH, BROAD LOW	Fail
R72B10	CG9652	AMMC, LH, PCB, SOG	Fail
R72C01	CG9652	AMMC, FSB, PCB, OL, SOG, MB	Fail
R72B03	CG9652	PI, PROW, AL, ANTLER	Increased sleep
R72B02	CG9652	MB, OL	Rescue
R72B08	CG9652	MB, OL, AL	Rescue

“Sites of Primary Expression” is operationally defined as neuroanatomical regions that display highest expression by review of confocal stacks from Fly-Light expression studies. Abbreviations for brain regions expressing Gal4: EB, ellipsoid body; LH, lateral horn; SOG, subesophageal ganglion; MB, mushroom body; AL, antennal lobe; FSB, fan-shaped body; NOD, noduli; BROAD LOW, indicates a broad, non-specific low expression of Gal4 in the brain; OL, optic lobe; AMMC, antennal mechanosensory and motor center; PCB, protocerebral bridge; PI, pars intercerebralis/median bundle.

### Sleep behavior

To assess sleep and activity of individual flies, the *Drosophila* Activity Monitoring System (DAMS; Trikinetics, Waltham, MA) was used. For each F1 progeny genotype, ∼40 3–5 d old males were collected in batches of 10 animals and stored overnight at 25°, and maintained on the same 12:12 LD cycle as for the genetic cross and stocks. The next day, 32 individual males were transferred via aspiration into DAMS monitor tubes that contained standard food. Tubes were then loaded into a DAMS monitor, 32 tubes of each genotype per monitor. Monitors were kept in DigiTherm CircKineticsTM incubators (Tritech Research) at 25° with a 12:12 LD cycle. Raw activity data were processed with the DAMFileScan program (Trikinetics) to sort the data into 1 and 30 min bins. The output files were analyzed using Sleep and Circadian Analysis Matlab Program (SCAMP) developed in the Griffith Lab (Waltham, MA).

### TrpA1 neuronal activation experiments

Individual Gal4 stocks were crossed to UAS-TrpA1 and reared at 23°. Transheterozygous F1 progeny (Gal4/UAS-TrpA1) were collected and reared in a similar manner as described above. Thirty-two individual animals of the three genotypes (Gal4/+, UAS-TrpA1/+, and Gal4/UAS-TrpA1) were placed in DAMS monitors at 23° at d 0, monitored for a baseline 24 hr day (d 1), and were shifted to 29° in either the entire 12 hr day period [Light Period (LP)] or 12 hr night period [Dark Period (DP)] in d 2 and the condition was repeated on d 3. Comparisons for change in sleep behavior were compared from d 1 (baseline) to d 2 and 3 within the given manipulation and observation period.

### Statistics

Data were analyzed using Prism Software (GraphPad). One-way ANOVA analysis with a Bonferroni Multiple Comparison Correction was used to determine differences between experimental genotypes (GAL4/DopR) and two controls: (GAL4/+) and (DopR/+) for all measures of sleep and activity. Identical comparative measures were employed for RNAi and TrpA1 experiments.

### Immunohistochemistry

Adult brains were dissected in 1 × PBS containing 0.05% Triton-X 100 (PBT), and then fixed overnight at 4° in 2% paraformaldehyde (EM Sciences) in PBT. They were washed for 5 × 10 min in 0.1% PBT, blocked for 1 hr in 0.1% PBT with 0.5% BSA and 5% normal goat serum, and then incubated with primary antibodies overnight at 4°. They were then washed, blocked, and incubated with secondary antibodies overnight at 4°. Washed tissues were mounted on glass slides in Vectashield (Vector Laboratories), small pieces of broken coverslips serving as posts, covered with a coverslip, and sealed. Primary antibodies were rabbit anti-DopR (1:1250, Kong *et al.* 2010), mouse anti-CD2 (1:50, Pierce), and mouse anti-Cherry (1:200, Biorbyt). Secondary antibodies were goat anti-rabbit Alexa Fluor 488 and goat anti-mouse Alexa Fluor 594 (Life Technologies). The tissues were imaged on a Nikon Eclipse Ti C1 Confocal System using 1 μm steps and a 40 × or 60 × oil-immersion lens.

### Data availability

The authors state that all data necessary for confirming the conclusions presented in the article are represented fully within the article.

## Results and Discussion

Given the broad innervation of dopaminergic neurons in the central brain and the potential for many different areas of complementary DopR regulation in the brain, we sought to both test new regions not associated with DopR function as well as regions previously characterized for DopR functions. We screened for rescue of the increased sleep of *DopR/+* mutants. Table 1 summarizes our screen results for FlyLightGal4 and Kaiser Gal4 lines associated with different regions of the *Drosophila* brain. Most lines screened failed to show significant differences from controls for sleep duration, used as primary criteria to characterize robust rescue and full restoration of DopR function. Although the ellipsoid body and noduli of the central complex express high levels of DopR protein (Kong *et al.* 2010; Lebestky *et al.* 2009), these structures do not appear to be related to DopR function in sleep. Additionally, DopR is normally expressed in the optic lobe (OL), and restoration does not appear to modulate the sleep phenotype. Similar to known results from the Kume lab (Ueno *et al.* 2012) that show that the dorsal FSB influences dopaminergic regulation of sleep, the c5-Gal4 line displays rescue of sleep phenotypes (Table 1). However, it should also be noted that this Gal4 line also expresses strongly in the mushroom body. Given the known relationship of the FSB to sleep regulation, we sought to pursue new regions in the brain that could influence dopaminergic regulation of sleep, such as the antennal lobe, subesophageal ganglion, and AMMC. However, these and other specific regions tested do not appear to influence sleep phenotypes (Table1).

**Figure 2:**
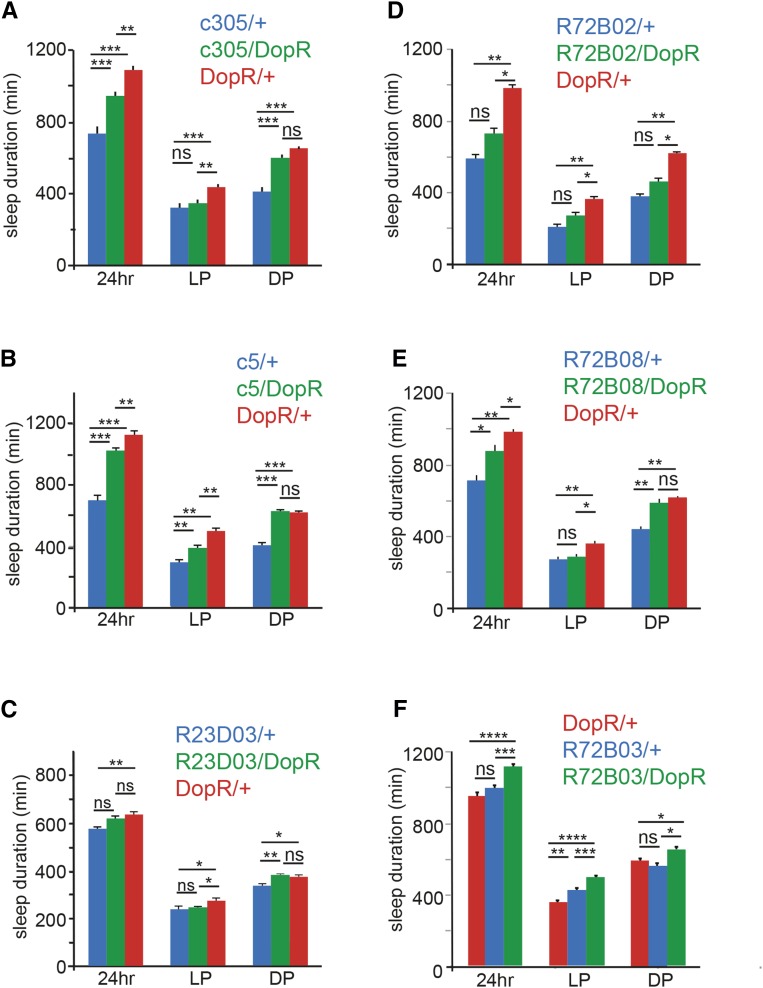
Rescue of day sleep by broad Gal4 expression including the mushroom body. (A–F) Genotype comparison of sleep duration for rescue (blue: Gal4/+, green: Gal4/DopR, and red: DopR/+). Sleep duration measured in minutes for either 24 hr, light period (LP), and dark period (DP). (A) c305 Gal4 comparison. (B) c5 Gal4 comparison. (C) R23D03 Gal4 comparison. (D–F) Rescue of day sleep by Gal4 lines derived from DopR genomic enhancer elements (CG9652). (D) R72B02 Gal4 comparison. (E) R72B08 Gal4 comparison. (F) R72B03 Gal4 comparison. *n* = 32 for all conditions. Statistics by ANOVA and Bonferroni correction: * *P* < 0.05, ** *P* < 0.01, *** *P* < 0.001, and **** *P* < 0.0001. DopR, dopamine receptor; ns, nonsignificant.

**Figure 3:**
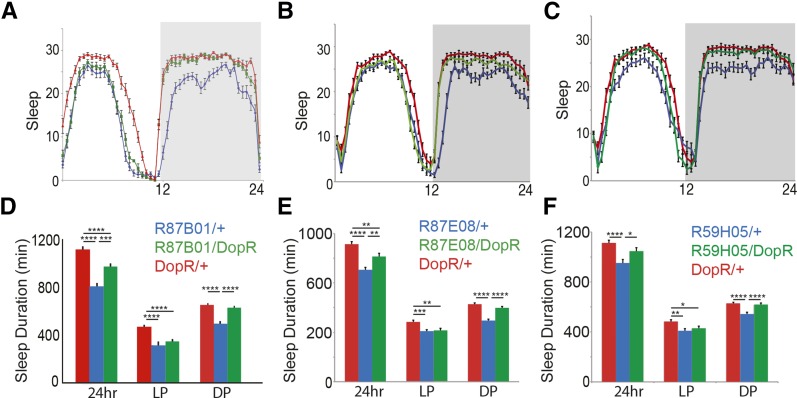
Rescue of day sleep by Gal4 expression in subsets of the mushroom body. (A–F) Genotype comparison of sleep and wake duration for rescue (blue: Gal4/+, green: Gal4/DopR, and red: DopR/+). (A–C) Sleep in 30 min bins represented for all genotype conditions over 24 hr period. (D–F) Sleep duration measured in min for either 24 hr, light period (LP), and dark period (DP). (A and D) R87B01 Gal4 comparison. (B and E) R87E08 Gal4 comparison. (C and F) R59H05 Gal4 comparison. *n* = 32 for all conditions. Statistics by ANOVA and Bonferroni correction: * *P* < 0.05, ** *P* < 0.01, *** *P* < 0.001, and **** *P* < 0.0001. DopR, dopamine receptor.

The mushroom body displays a nonuniform response when assessing DopR function. A number of Kaiser Collection Gal4 lines (201y, 117y, 43y, and 30y), that have been used by the *Drosophila* community for manipulation of mushroom body properties failed to display significant phenotypes when restoring DopR function. It should be noted that for two of these lines, the Gal4/+ control showed low activity/high sleep patterns that could obscure a clear shift in restoration of sleep behavior (data not shown). However, rescue was observed for c305a, c5, and four FlyLight Gal4 lines that express in the mushroom body: R23D03, R87E08, R87B01, and H05 (Figure 2 and Figure 3).

The Janelia Fly Light Collection is composed of Gal4 lines containing evolutionarily conserved transcriptional enhancer sequences derived from genes expressed in the adult *Drosophila* brain. Therefore, we also tested Gal4 enhancer lines that were specifically derived from the regulatory enhancer sequences of the DopR locus (CG9652), summarized in Table 2. Of the eight lines tested, two lines (R72B02 and R72B08) displayed significant rescue of the DopR sleep phenotype (Figure 2, D–F). These lines also express significantly in the mushroom body, and the R72B08/DopR rescue condition not only restores daytime sleep, but also nighttime sleep to wild-type levels. It should also be noted that two of the nonrescue lines tested also display expression in the mushroom body. Whether this negative data reflects low or insufficient Gal4 expression in the rescue condition, failure of overlapping expression with endogenous DopR circuits in the mushroom body, or potentially opposing roles for DopR dependent sleep regulation in subsets of the mushroom body and/or other Gal4-expressing regions within the given pattern is unknown. Independent of the observed relationship between DopR and the mushroom body as “prowake,” these experiments also potentially identified an opposing role for DopR as a “prosleep” modulator in the pars intercerebralis. When expressing DopR in the rescue condition, using the R72B03 Gal4 line, excessive sleep or inactivity is induced (Figure 2F).

In reviewing the lines tested, a common spatial determinant arising from our screening data that had the greatest effect on daytime sleep behavior appears to be the mushroom body (Figure 2 and Figure 3). The lines R87E08, R87B01, and R59H05 all restore daytime sleep, suggesting a new role for DopR in subsets of the mushroom body neurons (Figure 3). In all cases, the daytime sleep is restored, yet nighttime sleep is unaffected by DopR manipulation (Figure 3, D–F). Furthermore, a clear separation of DopR function in daytime sleep parameters *vs.* nighttime sleep parameters is also observed by close secondary parametric characterization of these spatially restricted lines (Supplemental Material, File S1).

**Figure 4:**
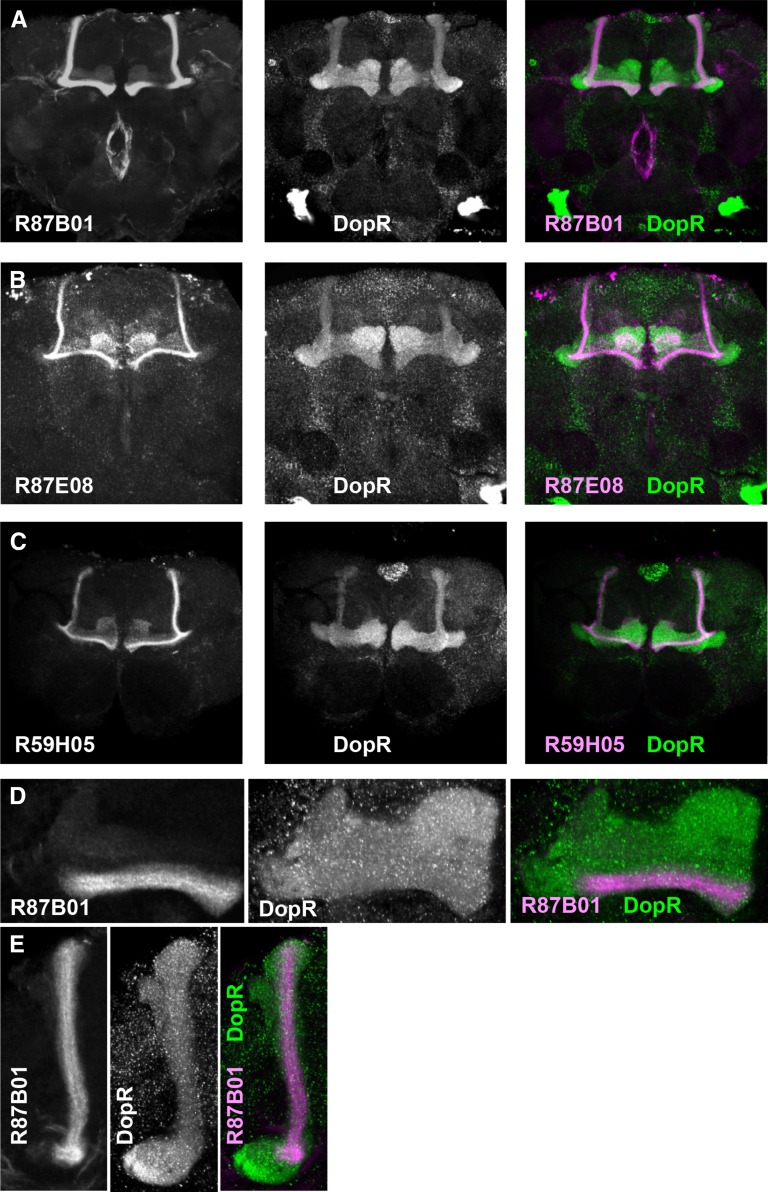
GAL4 and DopR expression patterns in the adult brain. Compressed confocal z-stacks of brains expressing UAS-CD2mCherry (magenta) in the indicated Gal4 patterns and immunostained for the DopR protein (green). (A) R87B01: 30 μm z-stack. (B) R87E08: 25 μm z-stack. (C) R59H05: 20 μm z-stack. (D and E) Higher magnification images of the β/γ (D) and α (E) lobes of the mushroom bodies of R87B01 > CD2mCherry brains. (D) 3 μm z-stack. (E) 10 μm z-stack. DopR, dopamine receptor; UAS, upstream activating sequence.

The Gal4 lines R87E08, R87B01, and R59H05 all display exceptional regional specificity (Figure 4). All three Gal4 lines show strong, restricted expression to subsets of neurons projecting within the α/β lobes of the mushroom body (Figure 4, A–C). In all cases, we observe overlap of DopR protein expression (green) and Gal4-driven CD2-mCherry within the mushroom body (magenta) (Figure 4, A–C). The R87B01 Gal4 line projects solely within α/β tracts of the mushroom body (Figure 4, A, D, and E), whereas R59H05 and R87E08 also show sparse expression in the mushroom body γ lobes (Figure 4, B and C).

**Figure 5:**
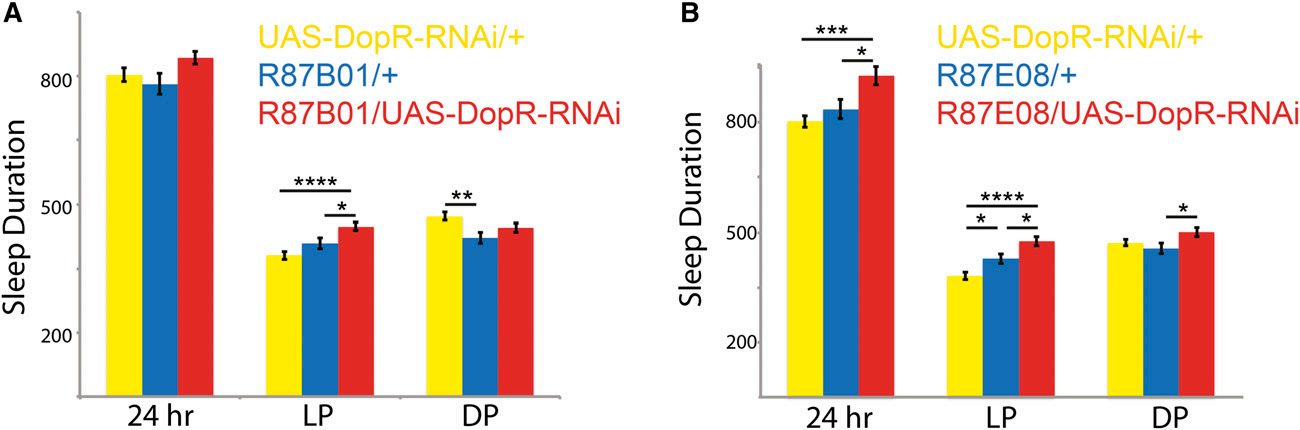
RNAi knockdown of DopR expression in subsets of mushroom body neurons. (A and B) Genotype comparison of sleep duration (yellow: UAS-DopR-RNAi, blue: Gal4/+, and red: DopR/+). Sleep duration measured in minutes for either 24 hr, light period (LP), and dark period (DP). (A) R87B01 Gal4 comparison. (B) R87E08 Gal4 comparison. *n* = 32 for all conditions. Statistics by ANOVA and Bonferroni correction: * *P* < 0.05, ** *P* < 0.01, *** *P* < 0.001, and **** *P* < 0.0001. DopR, dopamine receptor; RNAi, RNA interference; UAS, upstream activating sequence.

To confirm the requirement for DopR1 in the neuronal subsets of the mushroom body, we drove expression of an inducible UAS-DopR1-RNAi line (Keleman *et al.* 2012) in these cells and assessed effects on sleep (Figure 5). Both lines tested, R87B01 and R87E08, show increased daytime sleep, consistent with a specific requirement for DopR in the MB α/β lobes for daytime sleep regulation. As proof of principle, these experiments suggest that the screening methodology used with the *DopR^f02676^* allele is capable of positively identifying DopR^+^ neural circuits that influence sleep parameters.

**Figure 6:**
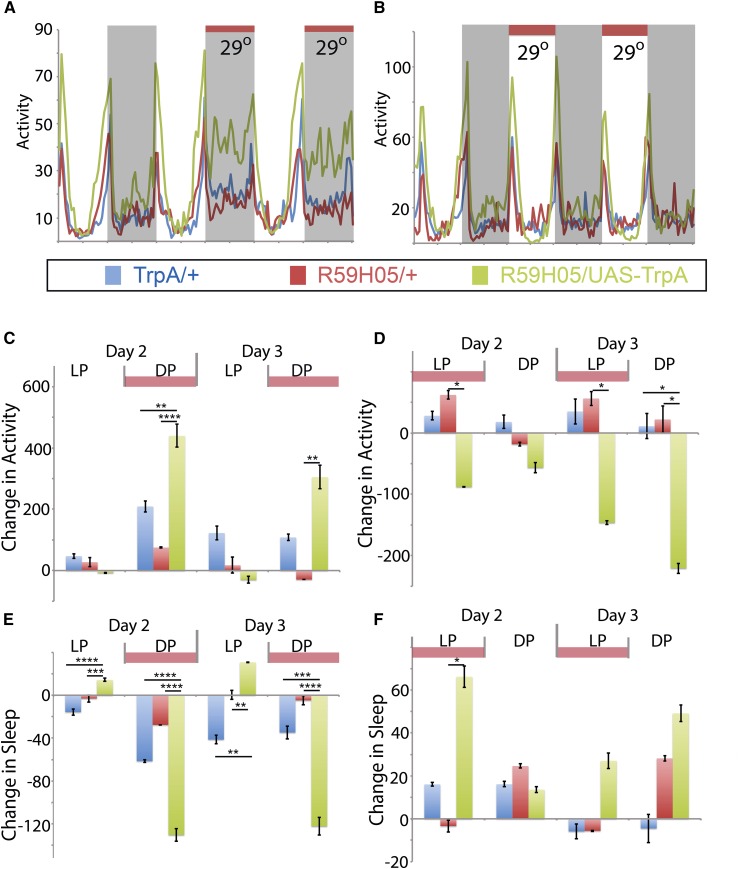
TrpA1 activation of R59H05-Gal4 expressing neurons. (A–F) UAS-TrpA1/+ (blue), R59H05/+ (red), and R59H05/ UAS-TrpA1 (green). (A and B) Activity plots for baseline (23°) and induced conditions (29°). (C–F) Change in activity (C and D) or sleep (E and F) on d 2 and 3 as compared to day 1 measured in min for either 24 hr, light period (LP), and dark period (DP). *n* = 28–32. Statistics by ANOVA and Bonferroni correction: * *P* < 0.05, ** *P* < 0.01, *** *P* < 0.001, and **** *P* < 0.0001. DopR, dopamine receptor; UAS, upstream activating sequence.

**Figure 7:**
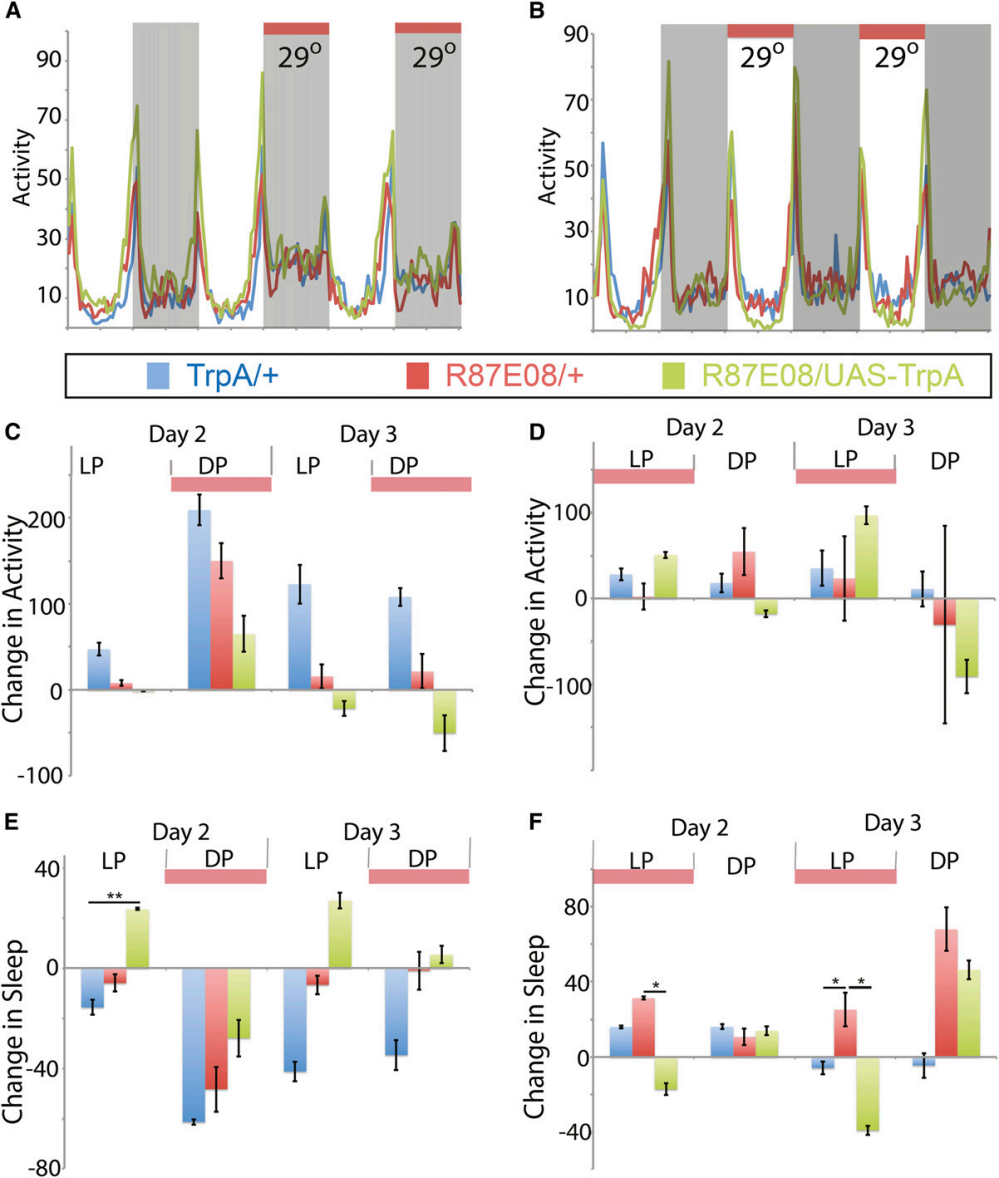
TrpA1 activation of R87E08-Gal4 expressing neurons. (A–F) UAS-TrpA1/+ (blue), R87E08/+ (red), and R87E08/ UAS-TrpA1 (green). (A and B) Activity plots for baseline (23°) and induced conditions (29°). (C–F) Change in activity (C and D) or sleep (E and F) on d 2 and 3 as compared to day 1 measured in min for either 24 hr, light period (LP), and dark period (DP). *n* = 28–32. Statistics by ANOVA and Bonferroni correction: * *P* < 0.05 and ** *P* < 0.01. DopR, dopamine receptor; UAS, upstream activating sequence.

Mushroom body Gal4 lines positively identified from the screen were also characterized by driving expression of UAS-TrpA1, to conditionally increase neuronal activity when elevating the environmental temperature (Figure 6 and Figure 7). Neuronal activation of R59H05 during the night period displays a significant positive shift in activity and loss of sleep (Figure 6, A, C, and E). However, activation of these neurons during daytime increases sleep during the day (Figure 6, B, D, and F). These phenotypes are different from the activation of R87E08 neurons, which show no phenotypic effect during nighttime activation (Figure 7, A, C, and E), but do show loss of sleep when activating the neurons during the day (Figure 7, B, D, and F). These data suggest a role for these neuronal subsets in regulation of sleep behavior, and R87E08 has a role consistent with the predicted relationship for DopR function as “prowake” during the day period. R59H05-Gal4 expressing neurons show a paradoxical “prosleep” behavior when activated during the daytime, in that peak dawn and predusk activities are elevated, but the “trough” of midday sleep is robust and an overall increase in sleep is observed (Figure 6B). This may suggest a more complicated modulatory relationship with dopamine in the phases of sleep regulation during the day, and the presence of additional molecular regulators in the overall function of these particular neurons.

Previous data suggested that DopR function in the whole mushroom body was not involved in sleep regulation, whereas the dorsal FSB plays a primary role (Ueno *et al.* 2012). The data were based on genetic restoration of DopR function using the OK107 Gal4 line that expresses broadly in the mushroom body. One source of difference is the number of lines in the mushroom body that were tested in our study. Additionally, OK107 expresses in multiple regions outside of the mushroom body, including the median bundle/pars intercerebralis, tritocerebrum, subesophageal ganglion, antennal lobe, OL, lobula, medulla, and transmedullary neurons (Morante and Desplan 2008). Additionally, our results for R72B03, which showed increased sleep levels due to expression of DopR in the median bundle/pars intercerebralis (Figure 2F), suggest the possibility that two opposing functions (prowake and prosleep) within OK107 may have partially obscured the role of DopR in the mushroom body. In contrast, the R87E08, R87B01, and R59H05 Gal4 lines show increased specificity within the mushroom body, and also mark a subset of the total MB neurons. Similar to recent studies that argue for microcircuit functions that individuate functions of a larger structure (Seidner *et al.* 2015; Sitaraman *et al.* 2015a), our data suggests that subsets of neurons in the mushroom body may mediate “prowake” regulation of daytime sleep.

Recent circuit activation experiments, utilizing UAS-TrpA1, have shown wake-promoting roles for the dopaminergic PPL1 and PAM subpopulations of neurons that **innervate subsets of** the α/β, α′/β′, and γ mushroom body (Sitaraman *et al.* 2015b). Our data suggests a complementary genetic requirement for DopR in subsets of mushroom body function. We do acknowledge the possibility that small contributions due to Gal4 expression outside of the mushroom body for any given line may contribute or act combinatorially with MB-Gal4 expression to influence sleep phenotypes. Thus, it will be informative to look at precise neural subsets within our positive circuit hits, using MARCM clones or Split-Gal4 reagents to further subdivide roles for our neuronal populations and potentially identify minimal sufficient neurons required for the phenotypes observed. Regardless of whether regions act combinatorially or individually, our study has operationally identified multiple new DopR^+^ neuronal subpopulations that appear to influence sleep in *Drosophila*. It will also be useful to further investigate a potential requirement for DopR function in the γ lobes, as opposed to other Dopamine receptor homologs that may be functioning in that structure. The data for R87E08-Gal4 and R59H05-Gal4 (Figure 3 and Figure 4) may support a potential role for *DopR* in the gamma lobe in “prowake” behavior during the day.

Our data collectively suggest that DopR in anatomical subsets of the mushroom body regulates daytime sleep with no significant impact on DopR-regulated night sleep patterns (Figure 3). This supports an interpretation of potential circuit separation of DopR-expressing neurons that regulate day *vs.* night sleep and arousal patterns, consistent with a large role for the dorsal Fan-shaped body in night sleep regulation (Ueno *et al.* 2012). Furthermore, distinctly different levels of cocaine-induced activity observed for wild-type animals during the day (low induced activity) *vs.* night (high induced activity) suggests potential separability between day and night that is nevertheless dependent in part on DopR function and may support spatially distinct circuits (Lebestky *et al.* 2009). Given the separability of day and night circadian oscillators (Grima *et al.* 2004; Stoleru *et al.* 2004) and the differential expression of arousal activities in the day and night (Ishimoto *et al.* 2012; Parisky *et al.* 2016), this data identifies a requirement for DopR in the mushroom body for daytime sleep and arousal regulation. Future immunohistochemical and double mutant analyses with *DopR* and previously identified day sleep modulators, such as sex peptide (Isaac *et al.* 2010), Ecdysone receptor, and DTS-3 (Ishimoto *et al.* 2012), may also be informative in better understanding the network of molecules involved in day sleep regulation.

## Supplementary Material

Supplemental Material
