# Supplementary material for: A Genetic Screen To Assess Dopamine Receptor (DopR1) Dependent Sleep Regulation in Drosophila
Source: G3 (Bethesda). 2016 Oct 18;6(12):4217–26. doi: 10.1534/g3.116.032136 (PMC5144989; doi:10.1534/g3.116.032136)
Supplement: Supplemental Material [file supp_6_12_4217__index.html]

A Genetic Screen To Assess Dopamine Receptor (DopR1) Dependent Sleep Regulation in Drosophila — Supplemental Material 

# A Genetic Screen To Assess Dopamine Receptor (DopR1) Dependent Sleep Regulation in *Drosophila*

## Supplemental Material for Jiang, *et al*, 2016

**Files in this Data Supplement:**

- File S1 - DopR function in day-time sleep parameters versus night-time sleep parameters. (.pdf, 3,327 KB)
