# Supplementary figures and images for: A Genetic Screen To Assess Dopamine Receptor (DopR1) Dependent Sleep Regulation in Drosophila
Source: G3 (Bethesda). 2016 Oct 18;6(12):4217–26. doi: 10.1534/g3.116.032136 (PMC5144989; doi:10.1534/g3.116.032136)

## Supplemental Data

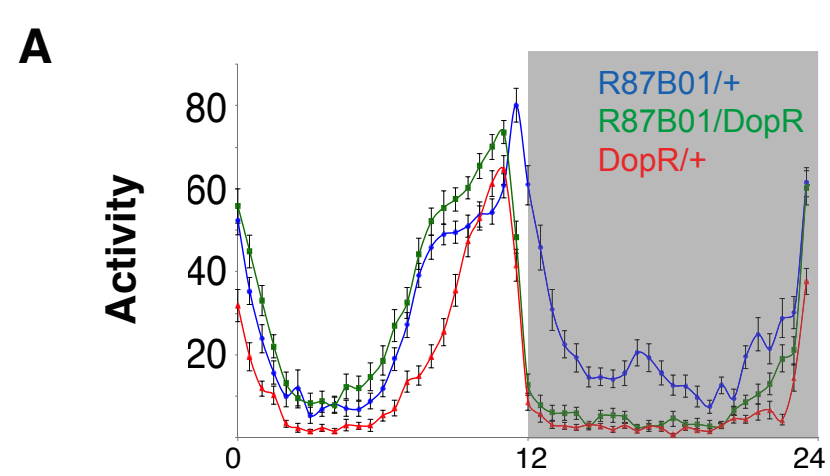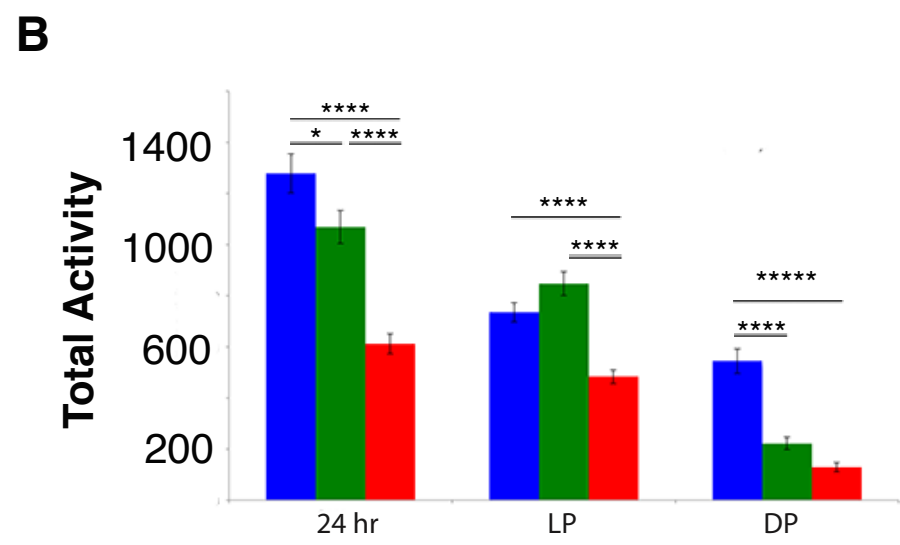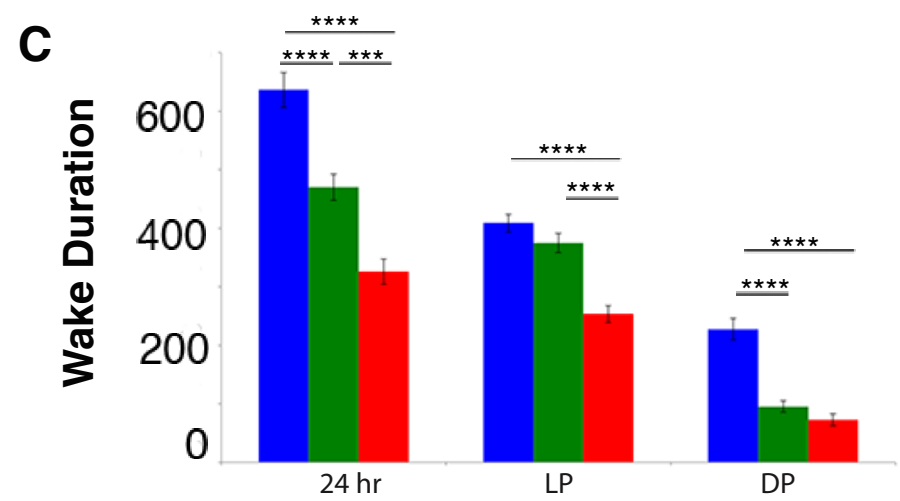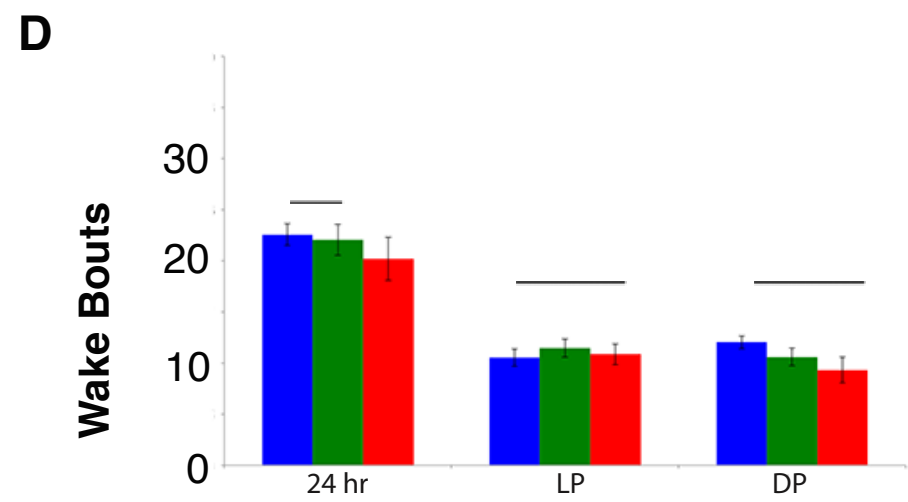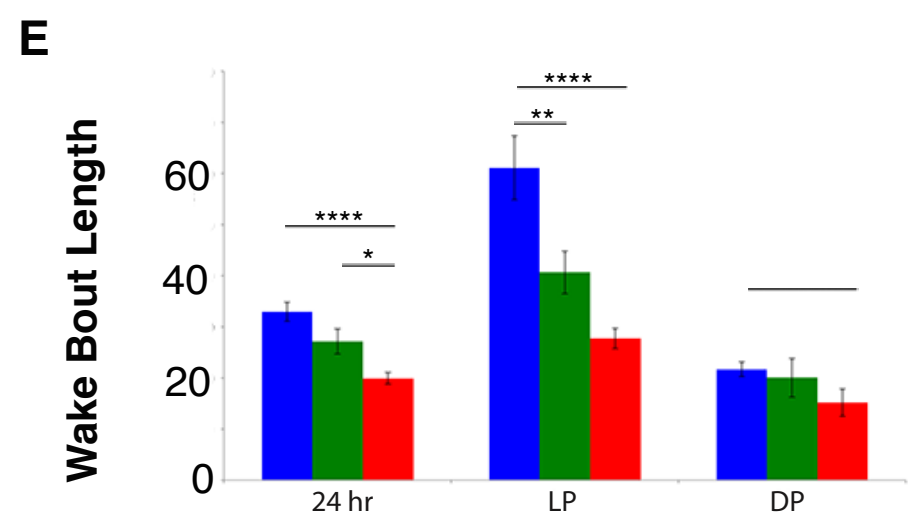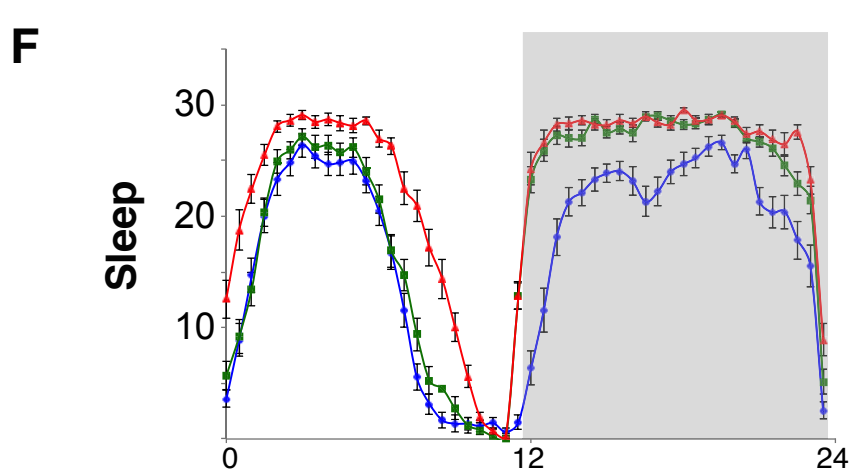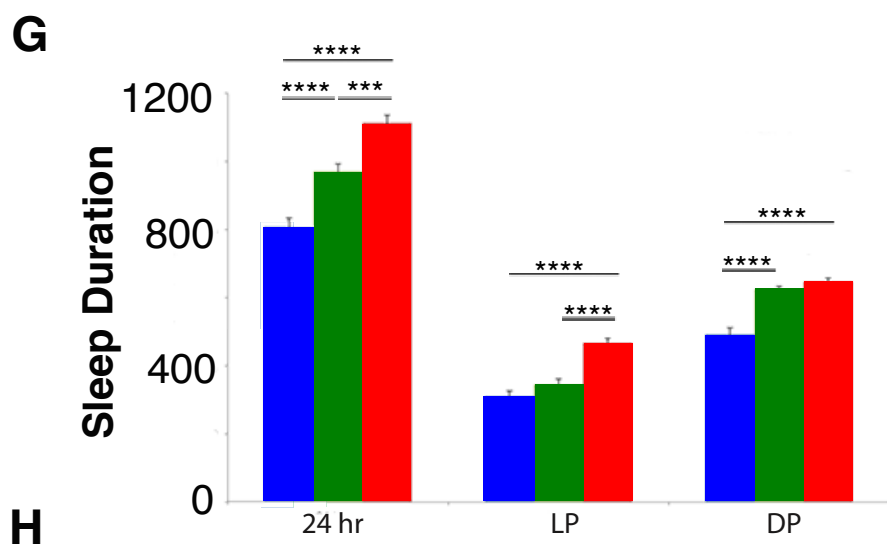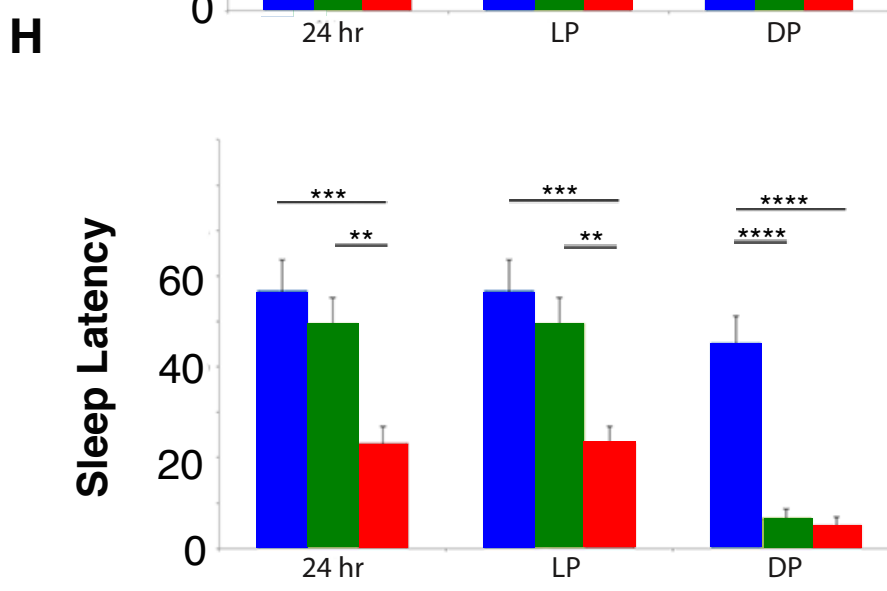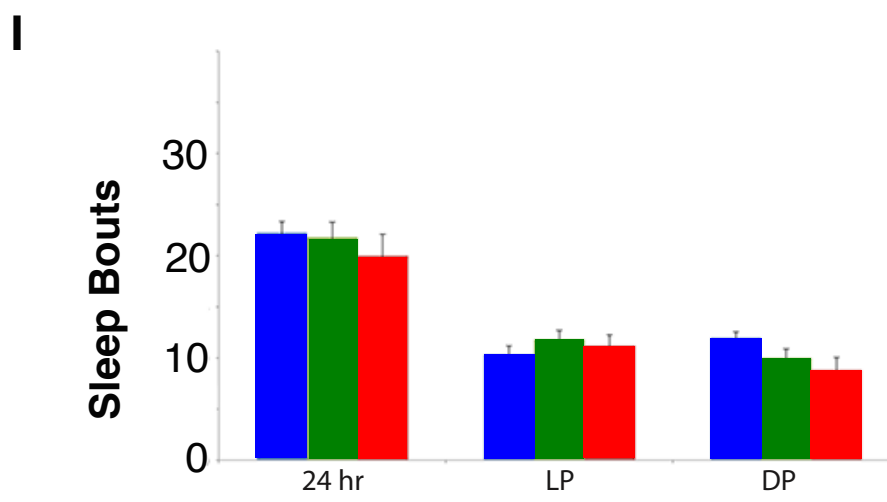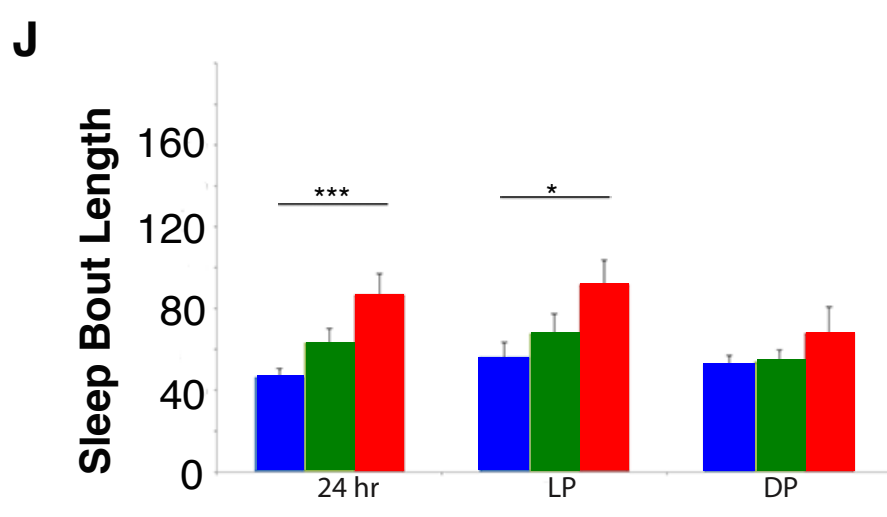

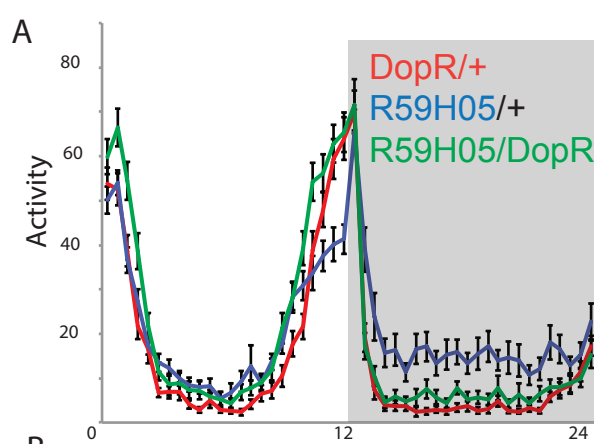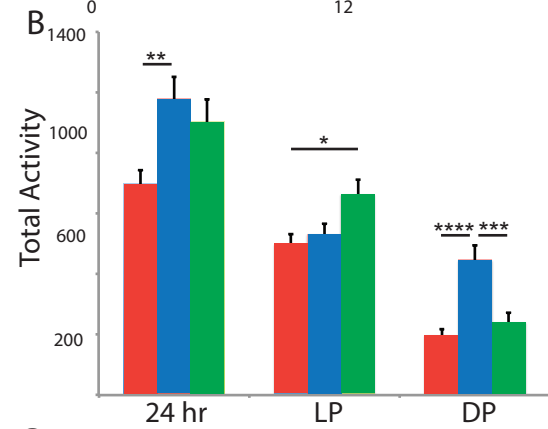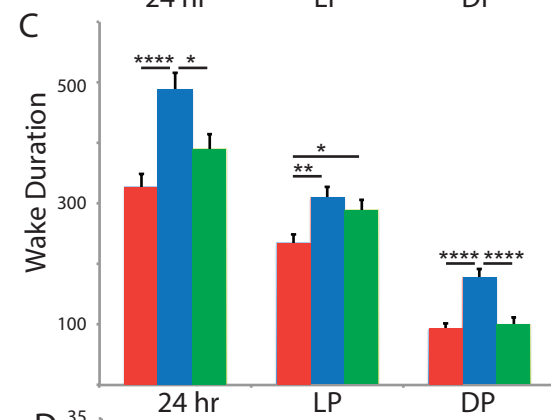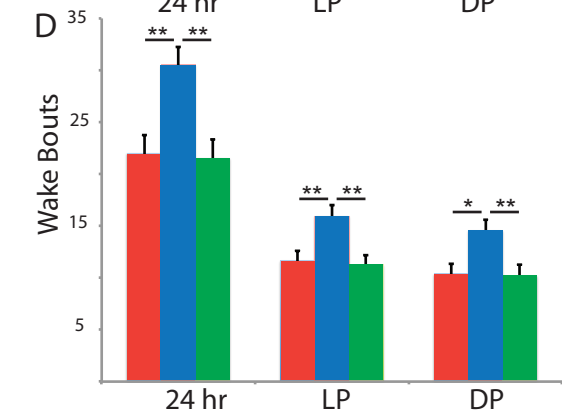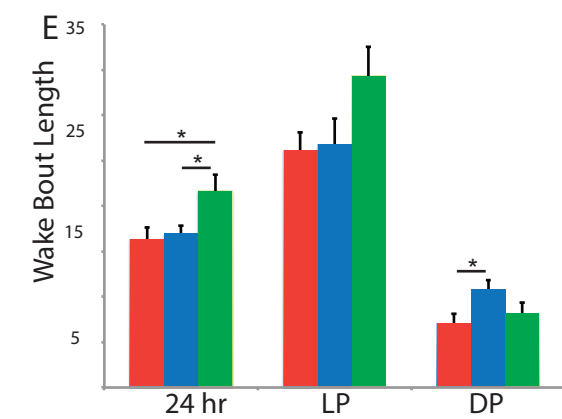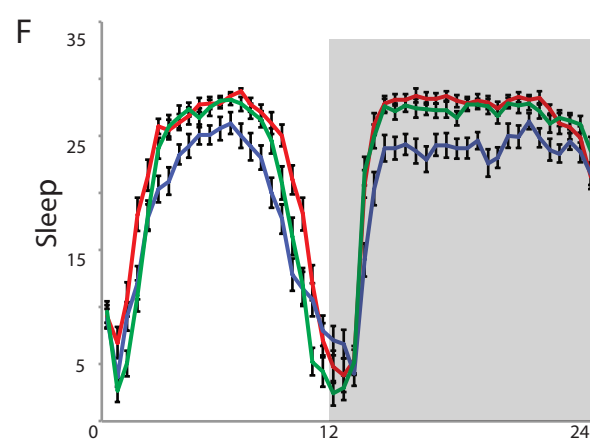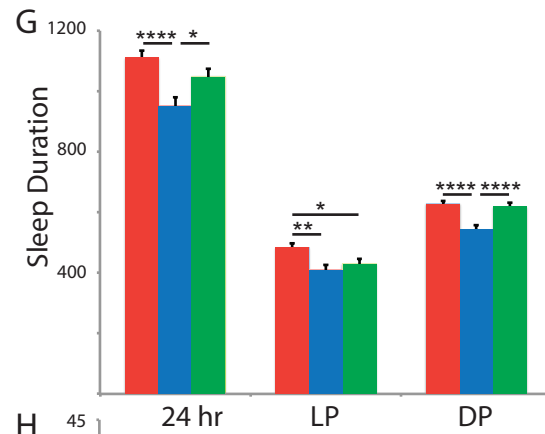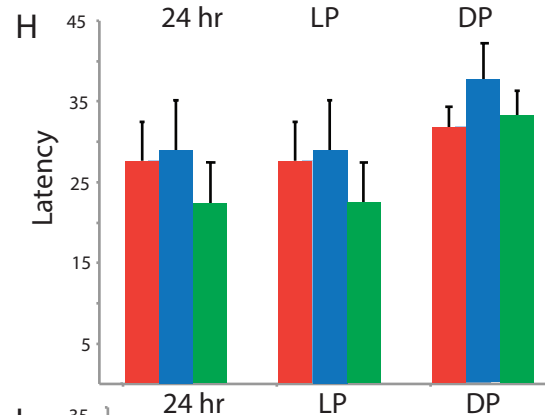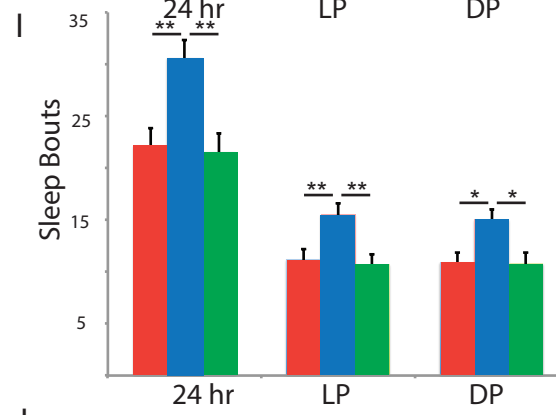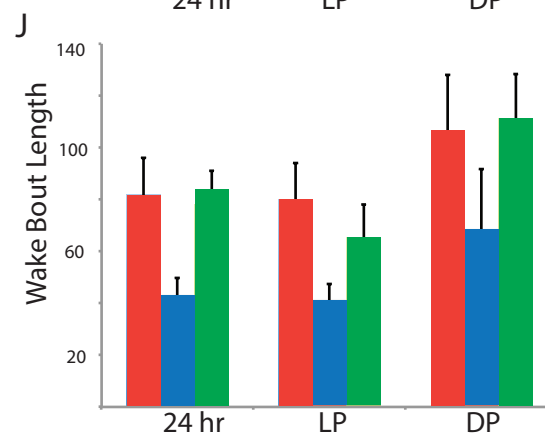

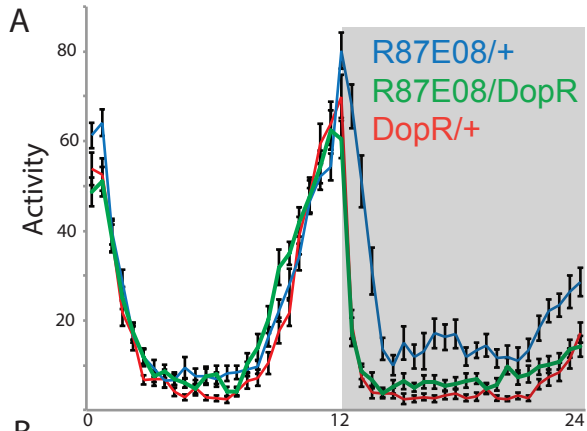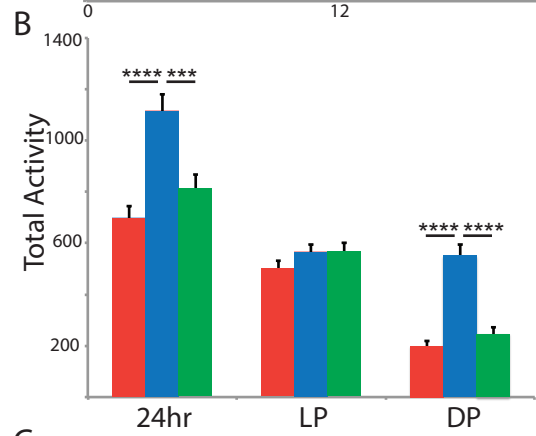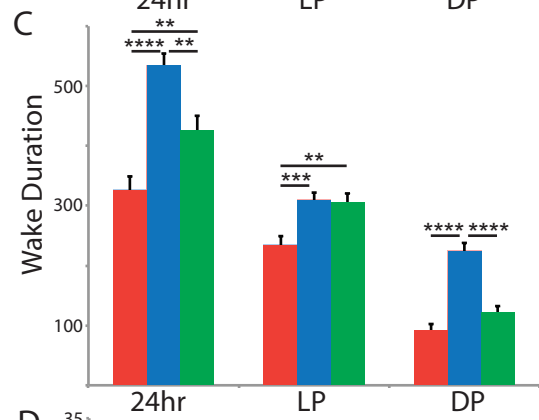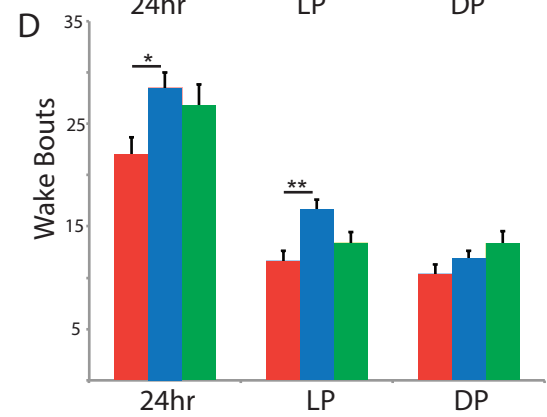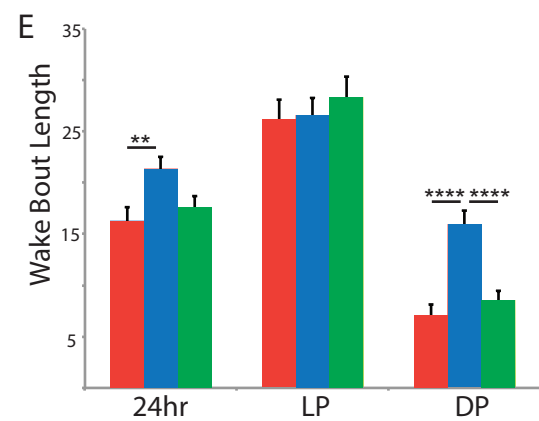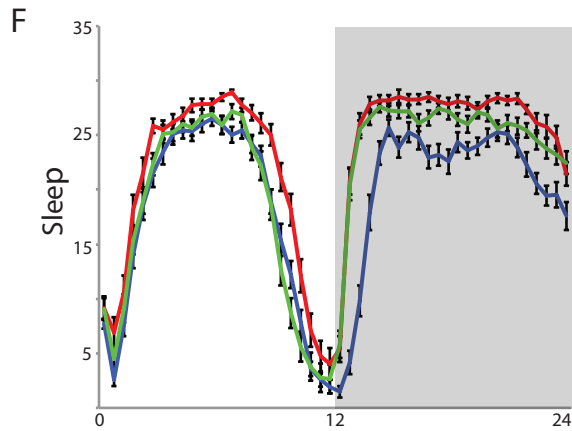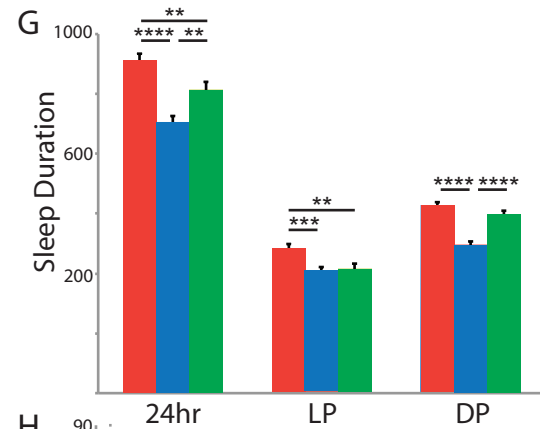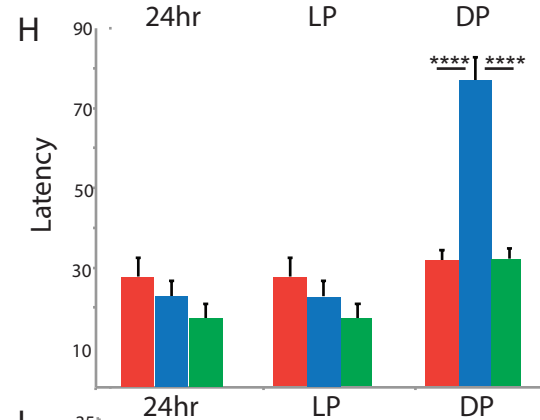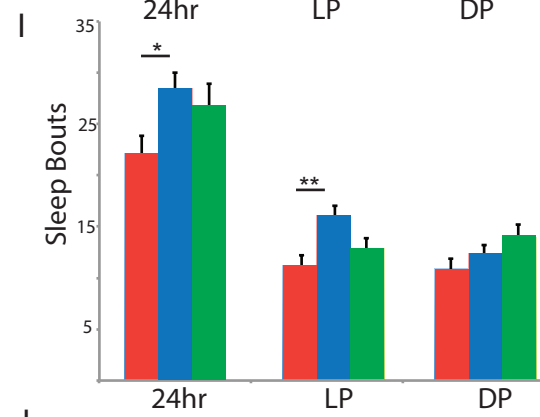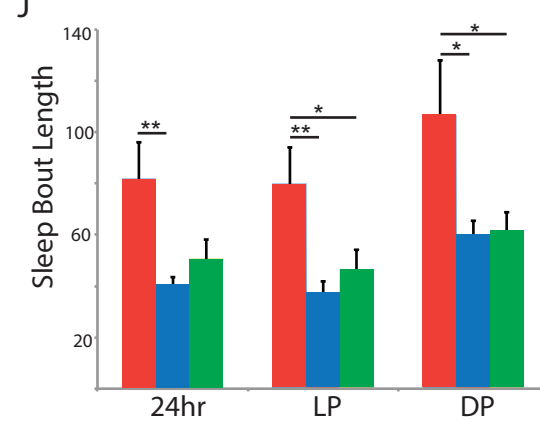

Supplement: Supplemental Material [file supp_g3.116.032136_FileS1.pdf]
